# The “true” 1-year incidence of dislocation after primary total hip arthroplasty: validation of an algorithm identifying dislocations in the Danish National Patient Register based on 5,415 patients from the Danish Hip Arthroplasty Register

**DOI:** 10.2340/17453674.2024.41064

**Published:** 2024-07-17

**Authors:** Lars L HERMANSEN, Thomas F IVERSEN, Pernille IVERSEN, Bjarke VIBERG, Søren OVERGAARD

**Affiliations:** 1 Department of Orthopedics, University Hospital of Southern Denmark, Esbjerg; 2 Department of Regional Health Research, University of Southern Denmark, Odense; 3 Department of Emergency Medicine, Gødstrup Hospital, Gødstrup; 4 The Danish Clinical Quality Program and Clinical Registries (RKKP); 5 Department of Orthopedics and Traumatology, Odense University Hospital, Odense; 6 Department of Clinical Research, University of Southern Denmark, Odense; 7 Department of Orthopedic Surgery and Traumatology, Bispebjerg Hospital; 8 Department of Clinical Research, University of Copenhagen, Denmark

## Abstract

**Background and purpose:**

Dislocations continue to be a serious complication after primary total hip arthroplasty (THA). Our primary aim was to report the “true” incidence of dislocations in Denmark and secondarily to validate a previously developed algorithm designed to identify THA dislocations in the updated version of the Danish National Patient Register (DNPR), based on data from the Danish Hip Arthroplasty Register (DHR).

**Methods:**

We included 5,415 primary THAs from the DHR performed from July 1 to December 31, 2019. Version 3 of the DNPR was launched in February 2019, and a combination of data from the DNPR and a comprehensive national review of 1,762 hospital contacts enabled us to identify every dislocation occurring during the 1st year after THA to determine the “true” 1-year incidence of dislocation. The results were presented as proportions with 95% confidence intervals (CI), and validation was performed by calculating sensitivity and predictive values.

**Results:**

The “true” 1-year incidence of dislocation was 2.8% (CI 2.4–3.3). Of these, 37% suffered recurrent dislocations during the follow-up period. Between-hospital variation ranged from 0.0% to 9.6%. The algorithm demonstrated a sensitivity close to 95%, while maintaining a positive predictive value of above 94%.

**Conclusion:**

The “true” 1-year incidence of dislocation of 2.8% is comparable to earlier findings, and large variation among hospitals continues to be evident. We have proven the algorithm to be valid in the latest DNPR (version 3), enabling it to be employed as a new quality indicator in future annual DHR reports.

Dislocation following total hip arthroplasty (THA) remains a serious complication that is accompanied by poor patient-reported outcomes and constitutes one of the leading indications for hip revision [1,2]. Several attempts have been made to reduce rates of post-THA dislocation with outcomes varying from 0% to 7%. The large variation is explained by the multifactorial causality of dislocation, which includes a combination of patient-, implant-, and surgery-related risk factors [3-6]. Moreover, clinical studies have often been limited to smaller cohorts, while larger register studies have a higher risk of overlooking complications not requiring open surgery.

To prospectively evaluate and reduce dislocation risk, it is necessary for larger registers to report dislocations more precisely and with less susceptibility to chance and for researchers to have access to updated and valid register data to ensure high national treatment quality. By combining register data and patient file reviews, we previously reported the “true” incidence of dislocation within 2 years after primary THA due to osteoarthritis (OA) in 2010–2014 to be 3.5%, with large variation between hospitals [7,8]. An algorithm based on applied diagnosis and procedure codes in the Danish National Patient Register (DNPR) was found to identify dislocations with high sensitivity and positive predictive value (PPV) [9].

Administrative systems are continuously changing, which unfortunately necessitates the performance of frequent validation studies to ensure accurate reporting in updated register systems. As a new version of the DNPR (version 3) was introduced in Denmark in 2019, we deemed it necessary to validate the previously developed algorithm in the updated register and at the same time to evaluate its effectiveness in a new cohort that included patients with diagnoses other than primary OA, which has not been done before.

Thus, the primary aim of our study was to identify the “true” incidence of THA dislocation based on all diagnoses and secondarily to assess the sensitivity and PPV of the algorithm in a cohort including multiple patient diagnoses in the new DNPR version 3.

## Methods

### Study design

This was a register study based on prospectively collected data reported to the Danish Hip Arthroplasty Register (DHR), the Danish Multidisciplinary Hip Fracture Register, and the DNPR. The study was conducted following the REporting of studies Conducted using Observational Routinely-collected Data (RECORD) guidelines [10].

### Setting and participants

All patients with primary THA performed at both public and private hospitals in Denmark in the 6-month period from July 1 to December 31, 2019, with any diagnosis and irrespective of previous hip surgery, were included in the study. Follow-up ended 1-year post-surgery or in the event of hip revision, death, or migration, whichever occurred first. This study took place before the COVID-19 pandemic. Hip revisions were excluded, as were any contralateral THAs during the inclusion period to avoid dependency among the observations [11]. The posterior approach is employed in 97% of THAs in Denmark [2].

### Outcomes and variables

THA dislocation was the primary outcome in this study. We define the “true” extent of this complication as a combination of data regarding every postoperative hospital contact from the DNPR, which holds a completeness of 99.7% [12,13], accompanied by a comprehensive, nationwide review of patient files. Dislocation required a hospital contact and was defined as the complete displacement of the femoral head from the acetabular cup, verified by radiographs and requiring reduction by any medical staff under local or general anesthesia. The correct International Classification of Diseases (ICD)-10 diagnostic code for THA dislocation in Denmark is widely accepted to be DT84.0A (Mechanical complication of internal joint prosthesis, hip). The Danish version of the Nordic Medico-Statistical Committees (NOMESCO) Classification of Surgical Procedures (NCSP) is currently the standard procedure code system in Denmark. In this system, the correct code for reduction is KNFH2* (reduction of dislocated joint prosthesis in the hip; *either closed, arthroscopic, or open).

Other codes included in the algorithm were the ICD-10 code DS73.0 (dislocation in native hip) and the NCSP code KNFH0* (reduction of the dislocated native hip joint; *either closed, arthroscopic, or open) [9].

The laterality feature in the DNPR is unique and permits a distinction between hips in patients with bilateral THAs. However, if a patient with bilateral THAs dislocates the left hip and presents with a bruised right knee, both sides (right and left) appear in the DNPR, and the laterality of the dislocation becomes uncertain. The algorithm incorporates a distinction between hospital contacts with or without known laterality [9].

### Data sources and linkage

Because of the unique 10-digit social security number (CPR number) assigned to all Danish citizens, identification and cross-matching to any national register is possible. This study thus included data from the DHR, the Danish Multidisciplinary Hip Fracture Registry, the DNPR, the CPR register, and patient files.

Patient identification and demographic information (age and sex) and the surgical characteristics of the primary procedure and potential revisions were extracted primarily from the DHR, which demonstrates a completeness of nearly 98% [2,14]. To capture patients with acute femoral neck fracture treated with THA who were erroneously not registered in the DHR, data from the Danish Multidisciplinary Hip Fracture Registry was included. This register possesses a PPV of 100% for primary prosthetic replacement procedure codes [15].

Revision procedures are reported with a completeness of 93–95% in the DHR [2,14]. To increase the revision data’s completeness, the DNPR was additionally utilized to extract data using all hip revision codes. Time of death was obtained from the CPR register. The DNPR was the source of information regarding any unplanned contacts (including dislocations treated by closed reduction) with the Danish healthcare system during the 1-year follow-up period, including date, hospital, diagnostic code, and procedure code. This register is an administrative database containing information on all hospital contacts in Denmark with a completeness of 99.7% [12,13]. The new version of this administrative register was introduced in February 2019 and contained substantial changes compared with the earlier system that aimed to improve the documentation of the patient’s course through the hospital system across different departments [16]. In the new data model, the hospitalization index initiates a sequence of contacts related to the specific clinical problem. One of the most significant changes in the DNPR version 3 is the omission of the variable “patient type” (inpatient vs outpatient vs emergency room [ER]), which makes it challenging to establish whether a patient was managed in the ER and sent home, admitted to a hospital department, or treated in an outpatient setting only.

Finally, the cause of every contact was determined by a review of relevant patient files. Each hospital contact was assigned a unique record identification number, and the data was uploaded into a Research Electronic Data Capture (REDCap) database (Vanderbilt University, Nashville, TN, USA) designed for the study [17]. The reviewer confirmed the dislocation, date of dislocation, laterality, number of dislocations during each hospital admission, and date of any additional dislocations during the same admission. In the event of missing information from the patient files regarding the cause of the hospital contact, the hospital’s radiographic database on the date of admission was consulted to confirm the dislocation.

### Statistics

Incidences of dislocation were presented as proportions with 95% confidence intervals (CI). The algorithm was evaluated by calculating the sensitivity (i.e., the proportion of true positives out of all dislocations), specificity (i.e., the proportion of true negatives out of all not having a dislocation), and PPV (i.e., the probability that patients identified as having a dislocation in the algorithm truly had a dislocation) for the algorithm’s first 4 steps [9]. The primary focus of the analysis was step 4, as the results from this step are intended to serve as a quality indicator in the DHR. STATA version 18.0 (StataCorp LLC, College Station, TX, USA) was utilized throughout the study for statistical analysis.

### Ethics, registration, funding, artificial intelligence use, and disclosures

Approval to review patient medical records was granted by the Region of Southern Denmark (21/21330). Approval to store and handle health data was given by registration in the Region of Southern Denmark’s internal research database (21/21482). The heads of the local orthopedic departments approved the study and decided whether the research group could visit the hospital to perform a personal review of the patient files or an affiliated secretary could make copies of the relevant contacts in the patient files and forward the information to the research group by secured mailing system.

The Danish Clinical Quality Program and Clinical Registries (RKKP) covered the costs associated with DNPR data extraction from the Danish Health Data Authority as well as 4 weeks of research salary for the primary investigator (corresponding author). Artificial intelligence was not used in this study. None of the participating authors have any conflicts of interest to report. Complete disclosure of interest forms according to ICMJE are available on the article page, doi: 10.2340/17453674.2024.41064

## Results

**Figure 1:**
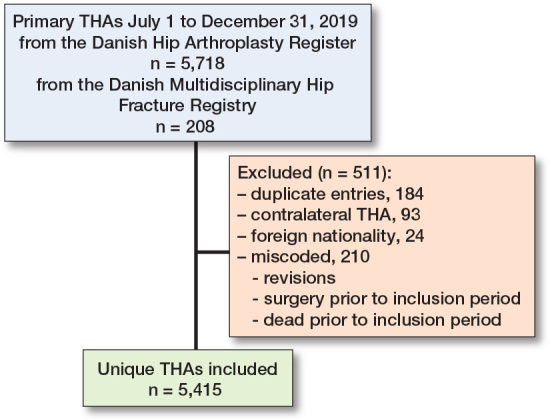
Flowchart of included and excluded patients.

5,718 THAs were registered in the DHR and 208 THAs were registered in the Danish Multidisciplinary Hip Fracture Registry; after data cleaning, the final study population consisted of 5,415 unique patients/THAs (Figure 1). The mean patient age was 69 years (range 13–101 years), and 55% were females. The 1-year follow-up was completed by 95% of the sample, while 2.5% underwent revision (any cause) and 2.5% died, yielding a mean follow-up of 353 days. During the first year, 0.7% were revised due to dislocations. The distribution of primary diagnoses is provided in Table 1. The cohort included patients from 42 public and private hospitals producing between 1 and 459 THAs.

**Table 1:** Diagnosis and indication for surgery reported to the DHR and incidence of dislocation

Diagnosis	Number of patients	Number of patients with dislocation	Incidence of dislocation % (CI)
Primary OA	4,472	113	2.5 (2.1–3.0)
Acute femoral neck fracture	269	12	4.5 (2.3–7.7)
Sequalae after proximal femur fracture	226	12	5.3 (2.8–9.1)
Hip dysplasia	155	3	1.9 (0.4–5.6)
Non-traumatic necrosis of the femoral head	104	4	3.8 (1.1–9.6)
Others	189	8	4.2 (1.8–8.2)
Metastases (44)			
Other (36)			
Rheumatoid arthritis (26)			
Acetabular fracture (23)			
Mb. Legg–Calvé–Perthes (23)			
Epiphysiolysis (9)			
Arthritis (other) (8)			
Congenital hip dislocation (8)			
Traumatic hip dislocation (5)			
Mb. Bechterew (4)			
Primary tumor (3)			

We manually reviewed 1,762 relevant hospital contacts and identified 240 contacts with 1 or more genuine THA dislocations, 131 contacts containing an already identified THA dislocation (e.g., new contact due to department/hospital transfer), 21 contacts with non-relevant dislocations (dislocation in either contralateral THA or ipsilateral hemiarthroplasty, revised to a primary THA on the day of admission), and 1,370 contacts without dislocation.

### Incidence of dislocation

We identified 243 dislocations distributed across 152 patients with 1 or more dislocations (maximum of 5), producing an overall “true” 1-year incidence of dislocation of 2.8% (CI 2.4–3.3), with a lower incidence in patients with primary OA and a higher incidence in patients with acute or previous hip fractures (Table 1). Between-hospital variation ranged from 0% to 9.6%, while the range was 0.7% to 6.7% in hospitals with a minimum volume of 100 patients. During the 1-year follow-up, 37% of the 152 patients suffered recurrent dislocations. The vast majority of patients were treated by closed reduction of the prostheses, and only 2 of the 243 dislocations required acute, open reduction (0.8%). One was due to a loose, rotated stem, and the other case was an anterior dislocation with failed closed reduction. Both occurred on the first postoperative day.

### Algorithm validation

**Figure 2:**
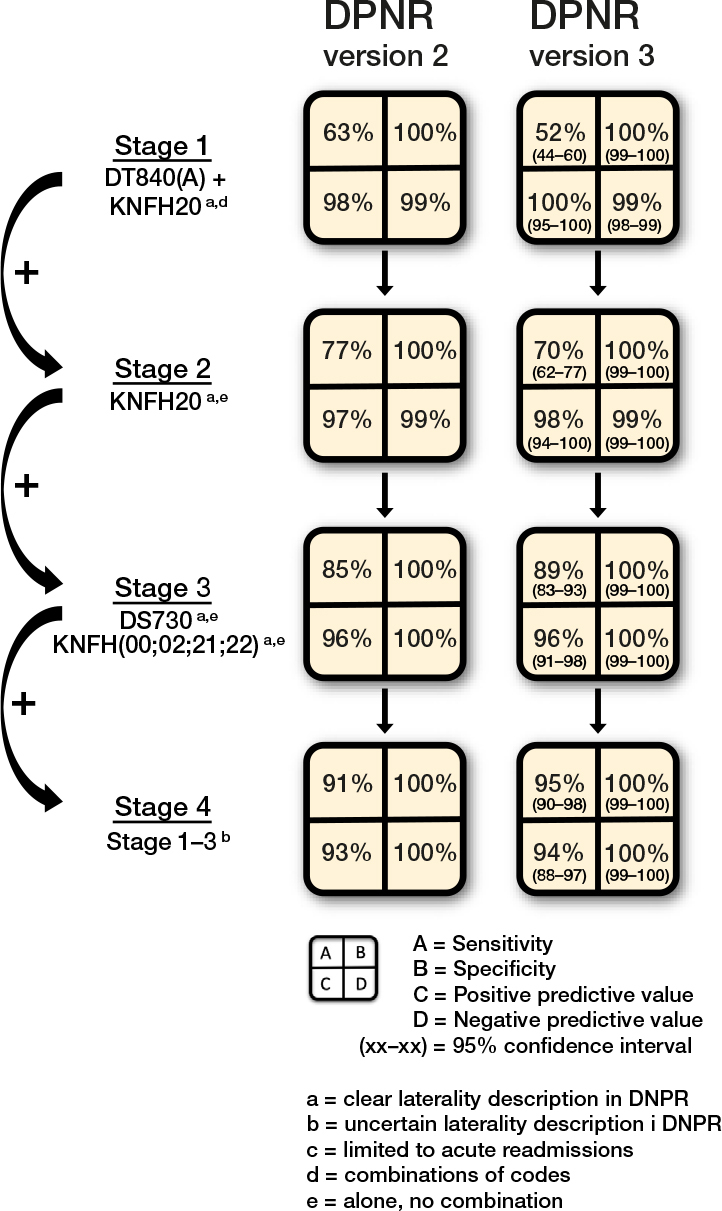
Sensitivity, specificity, positive and negative predictive values in different stages. For each step, additional codes are added to the previous step, thereby including more codes and increasing the sensitivity at the cost of decreased positive predictive value. The DNPR version 2 results were published in 2021 [9].

The stepwise approach in the algorithm revealed that if a combination of only the correct diagnosis and procedure codes were trusted in the DNPR version 3, only 52% (CI 44–60) of patients with dislocation within the first postoperative year would have been identified (Figure 2, stage 1). The incorporation of additional relevant and frequently applied codes resulted in a sensitivity close to 95% (90–98), while maintaining a PPV over 94% (85–95) (Figure 2, stage 4). Table 2 shows 2-by-2 data for each of the 4 stages.

**Table 2:** 2-by-2 table for each algorithm stage

Stage Patients (verified in patient files)	Patients (according to algorithm)	
	with dislocation	without dislocation
Stage 1		
with dislocation	79	73
without dislocation	0	5,263
Stage 2		
with dislocation	106	46
without dislocation	2	5,261
Stage 3		
with dislocation	135	17
without dislocation	6	5,257
Stage 4		
with dislocation	144	8
without dislocation	10	5,253

## Discussion

Our study aimed to identify the “true” incidence of dislocation after primary THA regardless of diagnosis and to assess the sensitivity and PPV of a previously published algorithm in a new cohort in the updated DNPR version 3. We found the overall “true” 1-year incidence of dislocation to be 2.8%, with a range of 0% to 9.6%. We were able to reproduce the previously demonstrated effectiveness of the algorithm by identifying dislocations treated by closed or open reductions with a high sensitivity of 95%.

We found the 1-year incidence of dislocation in patients with primary OA to be 2.5%, which corresponds well with the result from our 2010–2014 cohort, where dislocations were identified by an identical comprehensive and thorough review of patient files [8]. The primary outcome in the previous cohort was the 2-year incidence of dislocation, which was 3.5%, with approximately 80% of these occurring during the 1st year. Great care should be taken when comparing our results with studies from other countries or registries due to the high risk of underreporting. A recently published review employing international cross-country data from the last 6 decades concluded that the yearly dislocation rate had declined to as low as 0.7% between 2010 and 2020 [18]. Likewise, a large Medicare register study including data from 2010 to 2018 found a low 1-year dislocation risk of 1.6% in patients with OA [19].

Another important finding is that the patients revised due to dislocation comprise only 25% of the complete patient group with dislocations. This corresponds well with earlier reports and should be considered when interpreting dislocation results from arthroplasty registers with revision data only [8].

Several patient groups were included in this study, although only a minor sample suffered from diagnoses other than primary OA. Patients with acute femoral neck fracture and sequalae after previous hip fracture surgery had a 1-year incidence of dislocation of 4.5% and 5.3%, respectively. In contrast, a Medicare study including more than 10,000 patients treated with THA after acute femoral neck fracture between 2017 and 2019 found a lower risk of 2.9% within 1 year [20], and a Canadian register study of 4,612 THAs after acute femoral neck fracture reported only a 1.8% dislocation incidence 1 year post-surgery [21]. The large discrepancies are likely due to a combination of underreporting of complications and differences in surgical approach. HEALTH Investigators reported a dislocation rate of 4.7% in more than 700 patients with THA in a large, randomized trial comparing THA with hemiarthroplasty in patients with femoral neck fracture [22]. They followed each patient closely and did not rely solely on register data. However, their endpoint was complication rates at 2-year follow-up.

We previously reported large between-hospital differences in the dislocation burden [7]. This complication is indeed affected by several elements, including patient-, implant-, and surgery-related factors, which increases the risk of between-hospital variation due to differences in case mix, implant choice, and operative quality [8,23]. This large between-hospital variation was reproduced in the current study and emphasizes the need for continuous monitoring and local focus on addressing modifiable risk factors to reduce complication rates to levels achievable in comparable hospitals. Until now, it has been complicated and virtually impossible to gather usable data on dislocations treated by closed reduction, as national arthroplasty registers are limited to revisions only and national patient registers are highly dependent on clinicians applying the correct codes [24]. According to our previous findings, as few as 63% of patients with dislocation are captured when using a combination of the correct diagnosis and procedure codes in the DNPR [9], a rate that was confirmed by the current study’s finding that only 52% of patients with dislocation are identified from the correct diagnosis and procedure codes.

The present study is particularly strengthened by our ability to identify every postoperative contact with the Danish healthcare system and our comprehensive review of patient files, which enabled us to confirm each dislocation. Moreover, the Danish registries can distinguish laterality. Thus, we claim to present the “true” 1-year incidence of dislocation in a Danish setting. A limitation of our study was the small number of patients with acute fractures, which made us unable to perform a sound analysis of possible differences between the chosen implants, particularly between dual-mobility and fixed bearings.

### Conclusion

By combining data from the DHR and the DNPR, we have demonstrated the ability to capture up to 95% of all THA dislocations regardless of the indication for surgery while maintaining a high PPV. We have proven the algorithm to be valid in the latest DNPR (version 3), enabling its use to identify dislocations treated with closed/open reduction in a new quality indicator component of the annual DHR report. This will enable real-time reporting in individual orthopedic departments and hence the performance of local audits to explore potential areas for improvement, which may contribute to future reductions in the total number of patients experiencing dislocations after THA.

## Notes

LLH, BV, PI, and SO were responsible for the conception of the study; LLH was responsible for data analysis before and after review of patient files and coordinated the national review; TI assisted during the national review of patient files; LLH drafted the manuscript; BV and SO revised it critically for important intellectual content; and all authors approved the final version to be published.

The authors would like to thank the head of departments from the participating hospitals across Denmark. This study would not have been possible without their permission. They also thank the local heads of research and their secretaries, who they came across during this study and for their assistance during the national review of patient files.

Handling co-editors: Bart A Swierstra and Philippe Wagner

*Acta* thanks Bart G Pijls and Wierd P Zijlstra for help with peer review of this manuscript.
